# Prognostic implications of lactate dehydrogenase-to-albumin ratio in pediatric intensive care unit patients: a single-center prospective study

**DOI:** 10.1590/1984-0462/2026/44/2025380

**Published:** 2026-09-18

**Authors:** Aya Osama Mohamed, Yomna Ahmed Hosni

**Affiliations:** a Cairo University Children's Hospital, Faculty of Medicine, Pediatric Intensive Care Unit, Cairo, Egypt.; b Cairo University Children's Hospital, Faculty of Medicine, Diabetes, Endocrine and Metabolism Pediatric Unit, Cairo, Egypt.

**Keywords:** Critical illness, Organ dysfunction scores, Prognosis, Lactate dehydrogenases, Pediatric intensive care units, Doença grave, Escores de disfunção orgânica, Prognóstico, Relação lactato-desidrogenase, Unidade de terapia intensiva pediátrica

## Abstract

**Objective:**

To evaluate the lactate dehydrogenase-albumin ratio (LAR) as a mortality predictor in critically ill children.

**Methods:**

This study included 102 patients (aged 1 month to 14 years) admitted to the Pediatric Intensive Care Unit with critical illness, failure of one or more organ systems, over 6 months after exclusions. Initial labs included complete blood count, C-reactive protein, liver function tests, lactate-dehydrogenase (LDH), and albumin; subsequently, LAR was calculated. LDH, albumin, and LAR were correlated with the pediatric Sequential Organ Failure Assessment (pSOFA) score and mortality. Descriptive statistics, comparative tests, receiver operating characteristic (ROC) curve analysis, and logistic regression were performed.

**Results:**

Median age was 2 years, and 56.9% were male. Pulmonary causes were the main admission diagnosis (33.3%). Non-survivors had lower median albumin, higher median LDH and LAR levels, and higher rates of ventilation and inotrope use. The median LAR was higher in patients requiring ventilation and inotropes. LAR positively correlated with the pSOFA score and C-reactive protein levels. Logistic analysis showed LAR as a mortality predictor (AUC=0.82, cut-off 244.48, OR 1.004, 95%CI 1.002–1.006; p=0.001).

**Conclusions:**

In the Pediatric Intensive Care Unit, LAR could be used as a prognostic tool, especially in resource-limited countries, as it is accessible from routine laboratory tests.

## RESUMO

**Objetivo:**

Avaliar a relação lactato-desidrogenase/albumina (LAR) como preditor de mortalidade em crianças em estado crítico.

**Métodos:**

Este estudo incluiu 102 pacientes (com idades entre 1 mês e 14 anos) internados na Unidade de Terapia Intensiva Pediátrica com doença crítica e falência de um ou mais sistemas orgânicos, ao longo de seis meses após as exclusões. Os exames laboratoriais iniciais incluíram hemograma completo, proteína C reativa, testes de função hepática, lactato-desidrogenase (LDH) e albumina; em seguida, a LAR foi calculada. A LDH, a albumina e a LAR foram correlacionadas com o escore da Avaliação Sequencial de Falência Orgânica Pediátrica (pSOFA) e a mortalidade. Foram realizadas estatísticas descritivas, testes comparativos, análise da curva *receiver operating characteristic* (ROC) e regressão logística.

**Resultados:**

A mediana da idade foi de 2 anos, e 56,9% eram do sexo masculino. As causas pulmonares foram o principal diagnóstico de admissão (33,3%). Os pacientes que não sobreviveram apresentaram mediana mais baixa de albumina, mediana mais alta de LDH e de LAR, e taxas mais elevadas de ventilação e uso de inotrópicos. A mediana da LAR foi mais elevada nos pacientes que necessitaram de ventilação e inotrópicos. A LAR apresentou correlação positiva com a pontuação da pSOFA e os níveis de proteína C reativa. A análise logística mostrou a LAR como um preditor de mortalidade (AUC=0,82, ponto de corte 244,48, OR 1,004, IC95% 1,002–1,006; p=0,001).

**Conclusões:**

Na Unidade de Terapia Intensiva Pediátrica, a LAR poderia ser utilizada como ferramenta prognóstica, especialmente em países com recursos limitados, por ser acessível por meio de exames laboratoriais de rotina.

## INTRODUCTION

Recently, there has been significant progress in monitoring and in highly advanced interventions for managing critically ill patients, such as high-flow nasal cannula therapy, noninvasive ventilation, high-frequency ventilation, and extracorporeal membrane oxygenation (ECMO), but mortality remains an existing issue, particularly among those highly susceptible cases^1^ and in low-income countries, ranging from 6.7 to 51.1%.^2^ For that reason, early recognition and identification of children at high risk of mortality continue to be an urgent issue that needs to be addressed in pediatric intensive care units (PICUs).^3^

Numerous markers, including lactate dehydrogenase (LDH) and albumin, are broadly applied to assess the burden of illness and clinical outcomes in pediatric patients with critical illness.^3,4^

LDH is a key enzyme of glycolysis and has been identified as a prognostic factor for infection^3^ and linked to serious illnesses, involving sepsis.^5^

Serum albumin has been traditionally utilized to indicate patients’ nutritional status.^6^ Moreover, albumin is a negative acute-phase reactant that is utilized to predict sepsis outcomes.^7^ However, multiple conditions, such as malnutrition and liver cirrhosis, may also affect albumin levels. Consequently, albumin has been used as a composite indicator, such as lactate to albumin (LAC/ALB) ratio, C-reactive protein to albumin (CRP/ALB) ratio, and procalcitonin to albumin (PCT/ALB) ratio, rather than as a prognostic indicator alone.^8^

Moreover, several validated scoring systems, including the pSOFA score, pediatric risk of mortality (PRISM) IV, and pediatric index of mortality (PIM) 3, are broadly used to evaluate disease severity and prognosis in PICUs.^4^ However, the use of severity scores requires multiple variables, which is more complex than using single and simpler biomarkers that reveal the composite pathophysiology of life-threatening diseases in children, with reasonable accuracy for predicting mortality.^9^ Therefore, there is a growing interest in studying simple, rapid, and readily available laboratory markers. The LDH/albumin ratio (LAR), a novel symbiotic marker that evaluates nutrition and inflammatory status, as well as cellular damage, proved to show a relationship with unfavorable outcomes in several critical conditions, such as an increased risk of adverse outcomes among patients with ischemic stroke, as well as in-hospital mortality in patients with acute respiratory distress syndrome.^5,10^

There is evidence that LAR is strongly related to poor outcomes in cancer patients, including reduced cancer-specific survival, and has been identified as a prognostic factor for overall survival^11^ and for mortality in patients with lower respiratory tract infection^12^

Nevertheless, data on LAR and its prognostic factors are scarce in the pediatric population. The study's purpose was to assess the potential of LAR as a prognostic indicator of mortality and to ascertain its association with disease severity in critically ill children.

## METHOD

This prospective study was conducted among critically ill children admitted to a tertiary-care PICU of the Cairo University Children's Hospital, which provides advanced critical care. One hundred and two critically ill patients, defined by failure of one or more organ systems, out of 299 patients presenting to the PICU were enrolled in the study, with an age range from 1 month to 14 years over a six-month period. The study excluded 197 patients with incomplete records and those with comorbidities that affected albumin levels, such as chronic hepatic, renal, or gastrointestinal disorders, or malnutrition.

Upon admission to the PICU, demographic characteristics (age, gender), vital signs, primary diagnosis, and any associated comorbidities were assessed; a comprehensive examination was applied, and the severity of illness was assessed using the pSOFA score including respiratory (oxygenation parameters), cardiovascular (mean arterial pressure/vasoactive support), neurologic (Glasgow Coma Scale), hepatic (bilirubin), coagulation (platelet count), and renal systems (creatinine).^13^ The need for any vasoactive medications and/or mechanical ventilation, the length of PICU stay, system review, which refers to organ system involvement upon discharge or death, and mortality were documented. Laboratory tests were performed on the initial blood specimen, including complete blood count, C-reactive protein, liver function tests, coagulation profile, LDH, and albumin, after which LAR was determined.

Blood samples collected for LDH and albumin tests were immediately centrifuged, and the separated serum was analyzed and measured using the AU480 automated chemistry analyzer (Beckman Colter, USA) according to the manufacturer's instructions. Laboratory reference ranges for albumin: 3.8–5.4 g/dL and LDH: 90–190 U/L were those adopted by the clinical laboratory of Abo El-Reesh at Cairo University Children's Hospital.

The study protocol (N74-2024) was reviewed and approved by the Research Ethics Committee at Kasr Al Ainy Faculty of Medicine, Cairo University. Written informed consent was obtained from all enrolled patients and/or guardians. This study was conducted and reported in accordance with the Strengthening the Reporting of Observational Studies in Epidemiology (STROBE) statement.

An *a priori* sample size calculation was performed using OpenEpi, with an alpha level of 0.05, power of 0.95, an anticipated frequency of 50% among PICU patients, a design effect of 1, and accordingly, 92 patients were required to reject the null hypothesis. The average number of patients admitted to the PICU was 120 per six months. Tthe dropout rate was calculated as 10 patients, and the final sample after accounting for the 10% dropout was 102 patients. While there are prior studies estimating the LAR in PICUs, there is insufficient evidence to determine an expected frequency directly applicable to the PICU population under study. Therefore, a conservative expected frequency of 50% was used for calculating the sample size. The equation used to calculate the sample size was:


n=DEFF×N×p(1−p)[(d2/Z21−a2)×(N−1)]+p(1−p)

Where:

n=required sample size;

DEFF=design effect;

N=population size;

p=expected frequency of the outcome;

d=desired precision (margin of error); and

Z_1_−α/2=standard normal value corresponding to the selected confidence level.

All consecutive patients admitted to the PICU at Abu El-Reesh Hospital, Kasr Al-Ainy, Cairo University, during the study period who met the inclusion and exclusion criteria were included in the study.

The Statistical Package for the Social Sciences (SPSS) version 28 (IBM Corp., Armonk, NY, USA) was employed for data entry and coding. Quantitative data were summarized as mean, standard deviation, median, minimum, and maximum, while categorical data were expressed as counts and percentage. The non-parametric Mann-Whitney test was applied to compare quantitative variables,^14^ while the chi-square (χ^2^) test was utilized for categorical data. When the anticipated frequency was less than 5, Fisher's exact test was performed.^15^ The Spearman's rank correlation coefficient was calculated to assess the correlation between quantitative variables.^16^ The best cut-off value of significant markers to identify mortality was derived using receiver operating characteristic (ROC) curve analysis, selecting the optimal threshold based on the Youden index to maximize sensitivity and specificity. Univariate logistic regression analysis was conducted to evaluate LAR as a prediction of mortality. Multivariate logistic regression analysis was conducted to identify independent predictors of mortality. The variables included in the model were LAR, mechanical ventilation, and inotropic support. These variables were selected based on clinical relevance and/or a p<0.05. A p-value less than 0.05 was regarded as statistically significant.

## RESULTS

**Figure 1:**
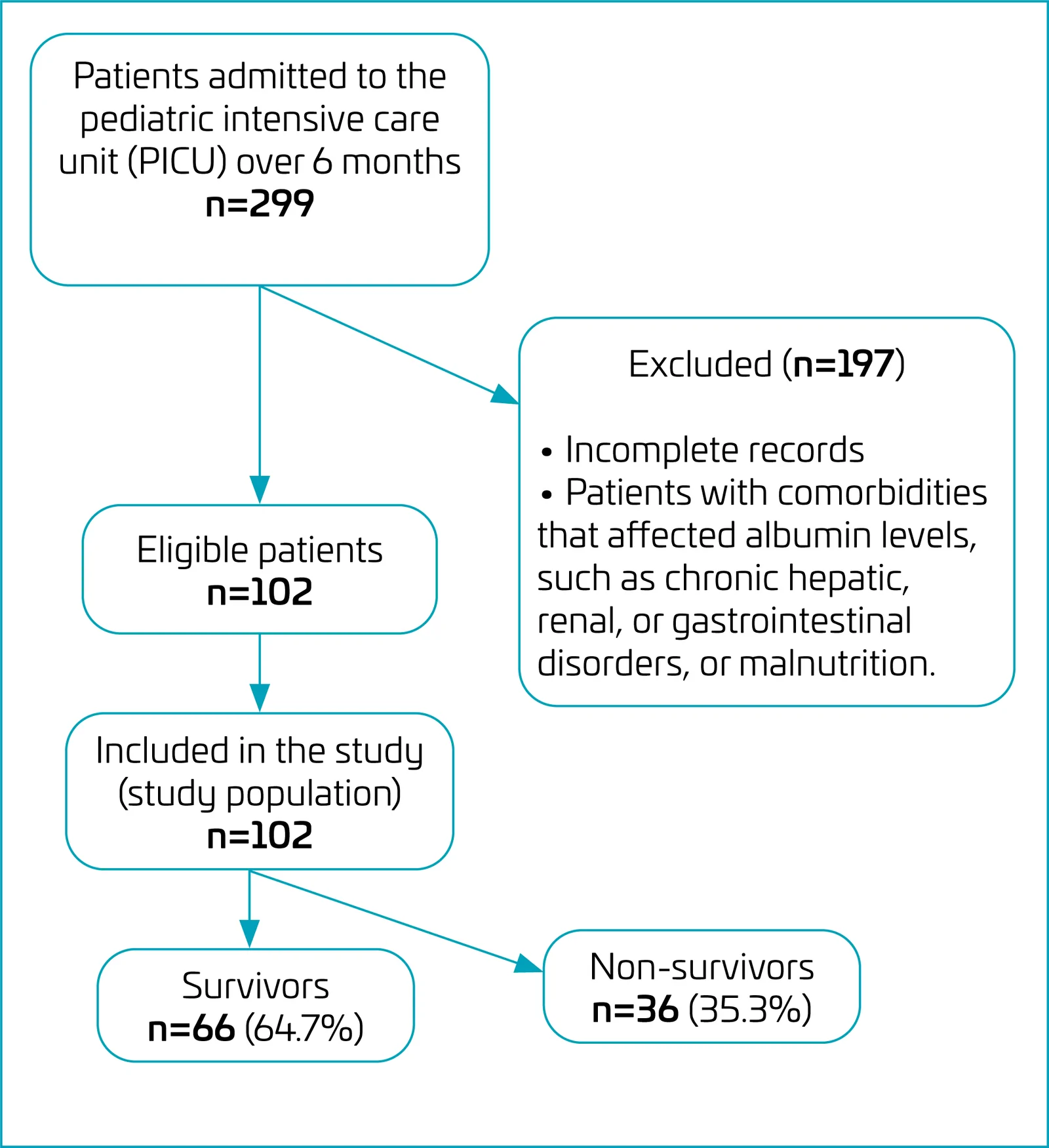
Flowchart of studied patients.

Source: Authors'own data.

The median age was 2 years (interquartile range [IQR] 0.6–6.0), and 58 (56.90%) were male. The clinical characteristics and laboratory investigations of the cohort are stated in Table 1 and Figure 1.

**Table 1:** Clinical and laboratory characteristics of the cohort on admission (survivors *versus* non-survivors).

	Cohort (n=102)	Survivors (n=66)	Non-survivors (n=36)	p-value
Age in years	2 (0.80–6)	2 (1–8)	1.5 (0.65–5.5)	0.580
Systolic blood pressure	90 (85–108)	97.5 (90–110)	90 (72.5–100)	0.028
Diastolic blood pressure	60 (52–67)	60 (55–70)	55 (50–62.5)	0.016
Temperature (°C)	37.5 (37.2–38.5)	37.5 (37.2–38.5)	37.7 (37.35–38.25)	0.171
Heart rate (beat/minute)	130 (117–150)	130 (120–150)	132.5 (114–150)	0.966
Respiratory rate	33.50 (25–40)	30.50 (25–40)	35 (26–41)	0.467
Capillary refill time (seconds)	3 (3–4)	3 (2–3)	4 (3–5)	<0.001
Glasgow Coma Scale	13.50 (12–15)	15 (13–15)	12 (8.5–13)	<0.001
pSOFA score	5 (3–10)	4 (2–6)	10.5 (5.0–15.5)	<0.001
LDH (U/L)	543 (345–1,056)	423.50 (307–667)	1,146.5 (552.5–1,978.5)	<0.001
Albumin (g/dL)	3.3 (2.8–3.8)	3.4 (3.0–3.9)	3.2 (2.8–3.5)	0.012
LAR	180.1 (102.4–310.6)	137.1 (92.7–203.7)	353.6 (187.3–690.3)	<0.001
PICU length of stay in days	8.5 (5–20)	8 (5–15)	10 (5.5–20.0)	0.474

Data are shown as median and interquartile range (IQR).

pSOFA: Pediatric Sequential Organ Failure Assessment; LDH: lactate dehydrogenase; LAR: lactate dehydrogenase-albumin ratio; PICU: pediatric intensive care unit.

Source: Authors'own data.

Respiratory conditions accounted for the majority of PICU admissions (33.30%), followed by neurological conditions and sepsis (each at 11.80%), then cardiac disease (4.90%), hepatic disease (3.90%), and renal disease (1%). The clinical severity profile for admitted cases is shown in Table 2. About 10.80% of the patients had neurological conditions, while 71 patients (69.60%) had no comorbidities. Of the patients, 54.20% were placed on inotropic support, 59.80% were ventilated, and 36 individuals (35.30%) were non-survivors. Regarding mortality, non-survivors had more abnormal findings on systems review than survivors (p<0.001; Table 2).

**Table 2:** Cohort severity profile during their stay in the pediatric intensive care unit.

		Cohort (n=102; %)	Survivors (n=66; %)	Non-survivors (n=36; %)	p-value
**Management during PICU stay**					
Ventilation in the PICU stay		55 (59.8)	21 (31.8)	34 (94.4)	<0.001
Inotropes in PICU stay		45 (54.2)	13 (19.7)	32 (88.8)	<0.001
Peritoneal dialysis		4 (3.9)	2 (3.0)	2 (5.6)	0.612
CRRT		4 (3.9)	1 (1.5)	3(8.3)	0.125
Plasma exchange		9 (8.8)	7 (10.6)	2 (5.6)	0.487
**PICU outcome**					
CNS review*	Abnormal findings	32 (32.0)	3 (4.5)	29 (85.3)	<0.001
CVS review*	Abnormal findings	30 (30.0)	0 (0)	30 (88.2)	<0.001
Pulmonary review*	Abnormal findings	30 (30.9)	0 (0)	30 (90.9)	<0.001
Hematological and metabolic review*	Abnormal findings	21 (22.8)	0 (0)	21 (75.0)	<0.001

Data are shown as count and percentage (%).

PICU: pediatric intensive care unit; CRRT: continuous renal replacement therapy; CNS: central nervous system; CVS: cardiovascular system.

* System review which refers to organ system involvement upon discharge, or death.

Source: Authors'own data.

The median values of LDH and LAR were elevated in both non-survivors and those with poor outcomes of systems review. In contrast, the median albumin value differed between survivors and non-survivors but did not reach statistical significance (Table 3).

**Table 3:** Comparison between lactate dehydrogenase, albumin, lactate dehydrogenase-albumin ratio, and mortality and system review.

		LDH (U/L)			Albumin (g/dL)			LDH/albumin ratio		
		Median	IQR	p-value	Median	IQR	p-value	Median	IQR	p-value
Mortality										
	Alive	423.5	307–667	<0.001	3.4	3.0–3.9	0.012	137.1	92.7–203.7	<0.001
	Dead	1146.5	552.5–1978.5		3.2	2.6–3.5		353.6	187.3–690.3	
CNS review										
	Normal	446.5	314.5–733.5	<0.001	3.4	2.9–3.9	0.275	144.5	95.1–221.4	<0.001
	Abnormal	842	500–1508.5		3.3	2.8–3.5		259.8	153.8–544.0	
CVS review										
	Normal	439	312–673	<0.001	3.4	3.0–3.9	0.097	142.3	92.8–204.9	<0.001
	Abnormal	1112	518–2050		3.3	2.7–3.5		334.6	182.5–699.5	
Pulmonary review										
	Normal	424	307–667	<0.001	3.4	3.0–3.9	0.067	140.7	92.7–203.7	<0.001
	Abnormal	1112	536–2050		3.3	2.6–3.5		334.6	191.9–699.5	
Hematological and metabolic review										
	Normal	443	312–717	<0.001	3.3	3.0–3.9	0.079	140.7	92.8–204.9	<0.001
	Abnormal	1155	569–2050		3.1	2.6–3.5		334.7	349.4–621.2	

Data are shown as median, interquartile range (IQR), and p-value.

LDH: lactate dehydrogenase, IQR: interquartile range; CNS: central nervous system, CVS: cardiovascular system.

* System review which refers to organ system involvement upon discharge or death.

Source: Authors'own data.

Ventilated patients had a higher median LAR (222.22; IQR 142.86–525.36) than non-ventilated patients (126.67; IQR 89.76–180.00; p<0.001). Likewise, LAR was elevated in patients on inotropes, with a median of 254.64 (IQR 145.16–525.36), compared with those not on inotropes with a median of 121.37 (IQR 92.70–175.56; p<0.001).

The correlations between LDH, albumin, and LAR with age, pSOFA score, and sepsis parameters are shown in Table 4.

**Table 4:** Correlation between lactate dehydrogenase, albumin, and lactate dehydrogenase-albumin ratio with the age and sepsis parameters of the cohort.

Cohort: n=102		LDH (U/L)	Albumin (g/dL)	LAR
Age in years				
	Correlation coefficient	-0.210	0.181	-0.244
	p-value	0.034	0.069	0.013
pSOFA score				
	Correlation coefficient	0.514	-0.264	0.535
	p-value	<0.001	0.007	<0.001
CRP				
	Correlation coefficient	0.179	-0.318	0.212
	p-value	0.072	0.001	0.033
Total lymphocytic count				
	Correlation coefficient	0.090	-0.171	0.129
	p-value	0.369	0.086	0.196

Data are shown as correlation coefficient, p-value, and sample size.

LDH: lactate dehydrogenase; LAR: lactate dehydrogenase-albumin ratio; pSOFA: Pediatric Sequential Organ Failure Assessment; CRP: C-reactive protein.

Source: Authors'own data.

LAR was positively correlated with PICU length of stay (r=0.040, p=0.690).

**Figure 2:**
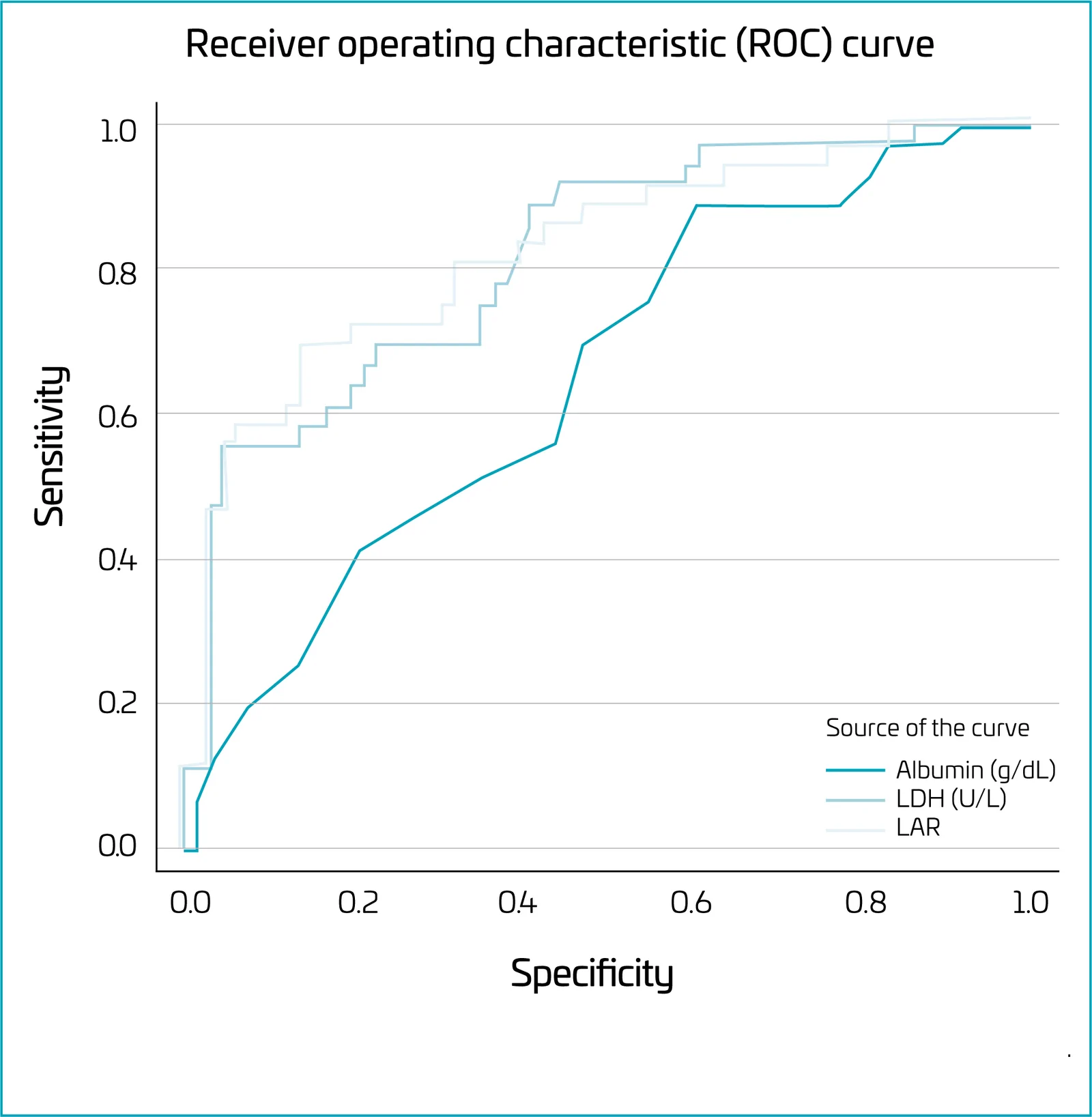
Receiver operating characteristic curve for lactate dehydrogenase, albumin, and lactate dehydrogenase-albumin ratio as predictors of mortality.

LDH: lactate dehydrogenase; LAR: lactate dehydrogenase-albumin ratio.

Source: Authors'own data.

The best cut-off values for LDH and LAR to predict mortality were 1078 U/L and 244.4762, respectively. Above this cut-off level, LDH and LAR were positive predictors of mortality (p<0.001; Figure 2).

The albumin cut-off value to predict mortality was 3.35 g/dL. Below this cut-off level, albumin was a positive predictor of mortality (p=0.007; Figure 2).

LAR was a significant predictor of mortality in the univariate logistic regression (p=0.001; odds ratio [OR] 1004; 95%CI 1002–1006) but became insignificant in the multivariate model after adjustment for potential confounders (p=0.780; OR 1.000; 95%CI 0.998–1.002).

## DISCUSSION

Early identification of children who need an advanced level of care and are at peak risk for mortality in the PICU has become essential.^3^ Recently, LAR has been associated with adverse outcomes in critically ill patients,^10^ its relationship with clinical outcomes was evaluated in the present cohort.

The most prevalent cause of admission was respiratory illness, which corresponds to the studies by Mohamed et al., Pisitcholakarn et al., and BinDahman et al.^17–19^ Of the 102 patients, 59.80% were ventilated, which was higher than that reported in two studies conducted by Mohamed et al.,^17^ and BinDahman et al.^19^ This may be related to the fact that the institutuion receives the most complicated cases at this tertiary center, requiring more advanced care. Among the patients, 54.20% needed inotropic support, which was slightly higher than in other retrospective studies conducted in a single facility.^18,20^ In addition, non-survivors had a higher rate of vasopressor use and a longer PICU stay, resembling the study by Wang et al.^20^

A high rate of mortality was observed, similar to that reported by BinDahman et al., who found 38.10%.^19^ However, this rate was higher than the study by Pisitcholakarn et al., who reported 14.10%.^18^ This may be related to the fact that this research was performed in a tertiary care facility, which receives the sickest and most vulnerable children.

This study demonstrated that non-survivors had unfavorable vital signs, including lower systolic and diastolic blood pressures and higher heart and respiratory rates, which were consistent with other studies.^8,20^

LDH was higher in non-survivors, which is consistent with numerous studies conducted by Mohamed et al., Jeon et al., and Algebaly et al.^17,21,22^ Additionally, hypoalbuminemia was observed in non-survivors, agreeing with various studies that reported lower albumin level was associated with worse clinical outcomes and higher mortality rates, supporting its role as a prognostic biomarker in critically ill pediatric patients.^7,8,20,21^

Similarly, LAR was higher in non-survivors, which is consistent with studies carried out by Jeon et al., Liu et al., and Guan et al., and Xia et al.^21,23,24^ These findings corroborate the study by Helliksson et al., who demonstrated that LAR was greater in patients with organ failure.^25^

LDH is a good marker of tissue hypoxia, the condition that is commonly seen in critically ill children^23^, whereas the pSOFA score is used to assess prognosis and severity of illness. A positive correlation was found between LDH and the pSOFA score, coinciding with the results by Mohamed et al. and Algebaly et al.^17,22^

Moreover, a negative correlation was observed between LDH and age, which differs from the findings of Mohamed et al.^17^ The difference may be explained by the fact that the present study covered all causes of PICU admission, whereas the study carried out by Mohamed et al. focused only on patients with sepsis.^17^

In this study, LAR positively correlated with the PICU length of stay, aligning with a study conducted in a university hospital in China, which showed that PICU length of stay rose steadily with increasing LAR.^23^

The LDH cut-off value for predicting mortality was higher than those reported by Algebaly et al. and Karlsson et al. (272 μL and 600 μL, respectively).^22,26^ This may certainly be related to the data of the present study that included all causes of PICU admission, encompassing a wide age range and a broad spectrum of diseases, in contrast to the study conducted by Algebaly et al., which included only patients with sepsis, and Karlsson et al., who considered only neonates.^22,26^

Comparing this study's LAR cut-off value as a positive predictor for mortality, to a study by Liu et al., LAR's cutoff value was 9.76, achieving the highest area under the curve (AUC) of 0.771^23^ A second study, conducted on 130 neonates admitted with neonatal sepsis, showed that the LAR cutoff value was 23.72.^27^ A third study, conducted among 583 adult patients to evaluate LAR as a prognostic factor in patients with serious infections admitted to the PICU, showed a cut-off value of 151.^21^ The wide discrepancy between the cut-off results may be because the first study included 8,782 pediatric patients, which is significantly larger than that of the present cohort. The second study was conducted on neonates, whereas the third included only patients with severe infections.

The sensitivity percentage observed in the present study was lower than those reported in the studies conducted by Liu et al., Xia et al., and Jeon et al. (72.30, 70.00, and 81.50%, respectively), indicating that LAR cannot be used as a good negative test in this population.^21,23,27^ However, the specificity percentage was higher than the previously mentioned studies (71.80, 88.20, and 41.20%, respectively), suggesting that LAR may be useful as a good diagnostic test.

The previous findings may reflect that both LDH and LAR could be used as positive predictive biomarkers for mortality. However, LDH alone - a simpler biomarker than LAR - may poorly reflect the complex pathophysiology of illness severity, which may limit its accuracy in predicting mortality^9^. Conversely, LAR, a peculiar composite biomarker that quantifies both cellular damage and nutritional/inflammatory status, has been shown to correlate with adverse outcomes.^5,10^

Serum albumin is considered a reflection of an acute phase response in patients with sepsis during their PICU stay.^7^ Consequently, a cut-off value was evaluated to determine its prognostic significance.

In the present study, the albumin cut-off level as a positive predictor of mortality was similar to that of a cohort study including all critically ill children upon admission, which reported low albumin levels associated with higher mortality, with a cut-off value of 3.40 g/dl.^28^ Similarly, a study conducted amongn adults presenting to the emergency department with sepsis reported a cut-off value of 3 g/dL.^8^ This finding points to the significance of albumin levels in predicting outcomes.

Univariate logistic regression analysis revealed that higher LAR was associated with mortality, aligning with other studies by Liu et al. and Xia et al. on pediatric patients and neonates, respectively.^23,27^ However, LAR became non-significant in the multivariate model after adjustment for potential confounders, in contrast to the findings of the large multicenter study conducted by Zhang et al., which demonstrated an independent association between LAR and mortality after multivariable adjustment.^29^ This may be related to the smaller sample size of the current study, population differences, and limited statistical power.

Moreover, LAR has a greater prognostic accuracy than either marker alone, as it provides a more comprehensive assessment of both the inflammatory and circulatory status of critically ill patients.^23^ In agreement with previous literature on the prediction of adverse outcomes, these findings support its potential role as a relatively rapid, readily available bedside biomarker, available through routine laboratory testing, with a practical advantage over more complex scoring systems.

This study is one of the few to evaluate the significance of LAR in predicting morbidity and mortality among patients admitted to the PICU.

This study has some limitations. First, it was conducted in a single center. Second, the relatively small sample size may limit the ability to detect modest associations between the studied biomarker and mortality. Third, LAR was calculated only initially at admission. Fourth, only univariate analyses were performed. Therefore, while LAR was associated with mortality, it cannot be concluded that it independently predicts outcomes after adjusting for confounding. Fifth, since the outcome's frequency exceeds 10%, OR may overestimate the true relative risks. Lastly, detailed clinical data for the excluded patients were unavailable and may be a source of selection bias. Further multicenter studies, including serial evaluation of LAR during the PICU stay and multivariate analyses, are needed to clarify the independent prognostic value of LAR.

In conclusion, the present study showed that the LAR was higher in non-survivors. Therefore, LAR could serve as a readily available biomarker for early prediction of mortality in children requiring advanced medical support, alongside other mortality prediction tools, especially in resource-limited countries, as LDH and albumin are available upon admission to the PICU.

## Data Availability

The database that originated the article is available with the corresponding author.

## References

[1] Ramnarayan P, Richards-Belle A, Drikite L, Saull M, Orzechowska I, Darnell R (2022). Effect of high-flow nasal cannula therapy vs continuous positive airway pressure following extubation on liberation from respiratory support in critically Ill children: a randomized clinical trial. JAMA.

[2] Molla MT, Endeshaw AS, Kumie FT, Lakew TJ (2023). The magnitude of pediatric mortality and determinant factors in intensive care units in a low-resource country, Ethiopia: a systematic review and meta-analysis. Front Med (Lausanne).

[3] Zou M, Zhai Y, Mei X, Wei X (2023). Lactate dehydrogenase and the severity of adenoviral pneumonia in children: a meta-analysis. Front Pediatr.

[4] Ari HF, Turanli EE, Yavuz S, Guvenc K, Avci A, Keskin A (2024). Association between serum albumin levels at admission and clinical outcomes in pediatric intensive care units: a multi-center study. BMC Pediatr.

[5] Zhang F, Zhang M, Niu Z, Sun L, Kang X, Qu Y (2024). Prognostic value of lactic dehydrogenase-to-albumin ratio in critically ill patients with acute respiratory distress syndrome: a retrospective cohort study. J Thorac Dis.

[6] Hansrivijit P, Yarlagadda K, Cheungpasitporn W, Thongprayoon C, Ghahramani N (2021). Hypoalbuminemia is associated with increased risk of acute kidney injury in hospitalized patients: a meta-analysis. J Crit Care.

[7] Takegawa R, Kabata D, Shimizu K, Hisano S, Ogura H, Shintani A (2019). Serum albumin as a risk factor for all the redundant references have been removed death in patients with prolonged sepsis: an observational study. J Crit Care.

[8] Chebl RB, Geha M, Assaf M, Kattouf N, Haidar S, Abdeldaem K (2021). The prognostic value of the lactate/albumin ratio for predicting mortality in septic patients presenting to the emergency department: a prospective study. Ann Med.

[9] Özel A, İlbeği EN, Yüce S (2025). Predictive value of initial lactate levels for mortality and morbidity in critically ill pediatric trauma patients: a retrospective study from a Turkish pediatric intensive care unit. Acute Crit Care.

[10] Chu M, Niu H, Yang N, Wang D, Liu Y, Mao X (2024). High serum lactate dehydrogenase-to-albumin ratio is associated with increased risk of poor prognosis after ischemic stroke. Clin Neurol Neurosurg.

[11] Peng RR, Liang ZG, Chen KH, Li L, Qu S, Zhu XD (2021). Nomogram based on lactate dehydrogenase-to-albumin ratio (LAR) and platelet-to-lymphocyte ratio (PLR) for predicting survival in nasopharyngeal carcinoma. J Inflamm Res.

[12] Lee BK, Ryu S, Oh SK, Ahn HJ, Jeon SY, Jeong WJ (2022). Lactate dehydrogenase-to-albumin ratio as a prognostic factor in lower respiratory tract infection patients. Am J Emerg Med.

[13] Matics TJ, Sanchez-Pinto LN (2017). Adaptation and validation of a pediatric Sequential Organ Failure Assessment score and evaluation of the Sepsis-3 definitions in critically ill children. JAMA Pediatrics.

[14] Chan YH (2003). Biostatistics102: quantitative data--parametric & non-parametric tests. Singapore Med J.

[15] Chan YH (2003). Biostatistics 103: qualitative data--tests of independence. Singapore Med J.

[16] Chan YH (2003). Biostatistics 104: correlational analysis. Singapore Med J.

[17] Mohamed NA, Youness ER (2021). Clinical role of serum lactate dehydrogenase assessment in critically ill pediatric patients with sepsis. Med J Cairo Univ.

[18] Pisitcholakarn V, Sunkonkit K, Reungrongrat S (2024). Incidence and factors associated with prolonged use of mechanical ventilation in pediatric intensive care unit in a single tertiary care hospital. PLoS One.

[19] BinDahman HA (2025). Mortality pattern and risk factors in pediatric ICU: a retrospective study at mukalla maternal and childhood hospital in Yemen (2021-2024). J Epidemiol Glob Health.

[20] Wang G, Liu J, Xu R, Fu Y, Liu X (2022). Lactate/albumin ratio as a predictor of in-hospital mortality in critically ill children. BMC Pediatr.

[21] Jeon SY, Ryu S, Oh SK, Park JS, You YH, Jeong WJ (2021). Lactate dehydrogenase to albumin ratio as a prognostic factor for patients with severe infection requiring intensive care. Medicine (Baltimore).

[22] Algebaly HAF, Abd-Elal A, El Kaffas R, Ahmed ES (2021). Predictive value of serum lactate dehydrogenase in diagnosis of septic shock in critical pediatric patients: a cross-sectional study. J Acute Dis.

[23] Liu M, Gou Y, Yang P (2025). Prognostic value of the lactate dehydrogenase-to-albumin ratio for predicting mortality in critically ill pediatric patients: a retrospective cohort study. Front Pediatr.

[24] Guan X, Zhong L, Zhang J, Lu J, Yuan M, Ye L (2024). The relationship between lactate dehydrogenase to albumin ratio and all-cause mortality during ICU stays in patients with sepsis: A retrospective cohort study with propensity score matching. Heliyon.

[25] Helliksson F, Wernerman J, Wiklund L, Rosell J, Karlsson M (2016). The combined use of three widely available biochemical markers as predictors of organ failure in critically ill patients. Scand J Clin Lab Invest.

[26] Karlsson M, Dung KT, Thi TL, Borgström E, Jonstam K, Kasström L (2012). Lactate dehydrogenase as an indicator of severe illness in neonatal intensive care patients: a longitudinal cohort study. Acta Paediatr.

[27] Xia X, Qiu S, Cheng X, Xie M, Zhou J (2025). Lactate dehydrogenase to albumin ratio as an independent factor for 28-day mortality of neonatal sepsis. Sci Rep.

[28] Kittisakmontri K, Reungrongrat S, Lao-Araya M (2016). Hypoalbuminaemia at admission predicts the poor outcomes in critically ill children. Anaesthesiol Intensive Ther.

[29] Zhang X, Liu J, Jiang R, Yang X, He W, Cao R (2026). Association between lactate dehydrogenase to serum albumin ratio and all-cause mortality among critically ill pediatrics: a retrospective cohort study. BMC Pediatr.

